# Combined effect of water loss and wounding stress on gene activation of metabolic pathways associated with phenolic biosynthesis in carrot

**DOI:** 10.3389/fpls.2015.00837

**Published:** 2015-10-15

**Authors:** Alejandro Becerra-Moreno, Mónica Redondo-Gil, Jorge Benavides, Vimal Nair, Luis Cisneros-Zevallos, Daniel A. Jacobo-Velázquez

**Affiliations:** ^1^Department of Biotechnology and Food Engineering, Centro de Biotecnologia-FEMSA, School of Engineering and Sciences, Tecnologico de Monterrey-Campus MonterreyMonterrey, Mexico; ^2^Department of Horticultural Sciences, Texas A&M UniversityCollege Station, TX, USA

**Keywords:** water stress, wounding stress, synergism in primary and secondary plant metabolism, phenylpropanoid metabolism, shikimic acid pathway, aromatic amino acid biosynthesis, lignification

## Abstract

The application of postharvest abiotic stresses is an effective strategy to activate the primary and secondary metabolism of plants inducing the accumulation of antioxidant phenolic compounds. In the present study, the effect of water stress applied alone and in combination with wounding stress on the activation of primary (shikimic acid) and secondary (phenylpropanoid) metabolic pathways related with the accumulation of phenolic compound in plants was evaluated. Carrot (*Daucus carota*) was used as model system for this study, and the effect of abiotic stresses was evaluated at the gene expression level and on the accumulation of metabolites. As control of the study, whole carrots were stored under the same conditions. Results demonstrated that water stress activated the primary and secondary metabolism of carrots, favoring the lignification process. Likewise, wounding stress induced higher activation of the primary and secondary metabolism of carrots as compared to water stress alone, leading to higher accumulation of shikimic acid, phenolic compounds, and lignin. Additional water stress applied on wounded carrots exerted a synergistic effect on the wound-response at the gene expression level. For instance, when wounded carrots were treated with water stress, the tissue showed 20- and 14-fold increases in the relative expression of 3-deoxy-D-arabino-heptulosanate synthase and phenylalanine ammonia-lyase genes, respectively. However, since lignification was increased, lower accumulation of phenolic compounds was detected. Indicatively, at 48 h of storage, wounded carrots treated with water stress showed ~31% lower levels of phenolic compounds and ~23% higher lignin content as compared with wounded controls. In the present study, it was demonstrated that water stress is one of the pivotal mechanism of the wound-response in carrot. Results allowed the elucidation of strategies to induce the accumulation of specific primary or secondary metabolites when plants are treated with water stress alone or when additional water stress is applied on wounded tissue. If the accumulation of a specific primary or secondary metabolite were desirable, it would be recommended to apply both stresses to accelerate their biosynthesis. However, strategies such as the use of enzymatic inhibitors to block the carbon flux and enhance the accumulation of specific compounds should be designed.

## Introduction

The application of postharvest abiotic stresses (i.e., wounding, UV-light radiation, modified atmospheres, exogenous phytohormones) has been proposed in recent years as an effective strategy to activate the secondary metabolism of plants leading to the accumulation of antioxidant phenolic compounds (Cisneros-Zevallos, 2003; Zhao et al., 2005; Schreiner and Huyskens-Keil, 2006; Jacobo-Velázquez and Cisneros-Zevallos, 2012). Among the different abiotic stresses studied, wounding is the most effective activating the phenylpropanoid metabolism and thus promoting a higher accumulation of phenolic compounds (Jacobo-Velázquez and Cisneros-Zevallos, 2012). Carrot has been used as a model system to study the effect of wounding stress on the phenylpropanoid metabolism (Howard and Griffin, 1993; Lafuente et al., 1996; Cisneros-Zevallos, 2003; Reyes et al., 2007; Jacobo-Velázquez and Cisneros-Zevallos, 2012; Surjadinata and Cisneros-Zevallos, 2012; Jacobo-Velázquez et al., 2015). Likewise, the application of wounding in combination with additional stresses (i.e., UV-light, phytohormones, and hyperoxia) has been reported as an approach to increase the wound-induced accumulation of phenolic compounds in carrots, and to manipulate their phenolic profiles (Surjadinata, 2006; Heredia and Cisneros-Zevallos, 2009a,b; Jacobo-Velázquez et al., 2011). Phenolic compounds that accumulate as a stress response in carrots are mainly hydroxycinnamic acids, such as chlorogenic acid (CHA), 3,5-dicaffeoylquinic acid (3,5-diCQA), and 4,5-dicafeoylquinic acid (4,5-diCQA).

Phenolic compounds in wounded plants are produced in part as a mechanism to support the biosynthesis of lignin, in order to prevent water loss (Whetten and Sederoff, 1995; Lulai and Corsini, 1998; Boerjan et al., 2003). Therefore, the accumulation of phenolic compounds observed in wounded plants has to be the result of a higher production rate when compared to the utilization rate for lignin biosynthesis (Jacobo-Velázquez and Cisneros-Zevallos, 2012). Likewise, phenolic compounds biosynthesis should also be dependent of the availability of L-phenylalanine (L-phe), the main amino acid used as precursor for the biosynthesis of phenylpropanoids (Hahlbrock and Scheel, 1989). L-phe is biosynthesized by the shikimic acid pathway, which is part of the primary metabolism of plants.

Water stress has been pointed out as a pivotal mechanism of the wound response in plants (Reymond et al., 2000). The effect of water stress on the primary and secondary metabolism of plants has been mainly analyzed in plants grown under drought conditions (Fan et al., 2006; Yang et al., 2006; Yoshimura et al., 2008; Sicher and Barnaby, 2012; Warren et al., 2012). However, the combined effect of water and wounding stress on the activation of the primary and secondary metabolism of plants has been underlooked. Moreover, when both stresses are evaluated independently, it has been reported that water and wounding stress exert a similar response at the gene expression level (Reymond et al., 2000). Therefore, storage conditions like relative humidity of stored fresh produce treated with wounding stress, a pre-requisite to produce fresh-cut fruits and vegetables, may be an important parameter to consider when the accumulation of phenolic compounds or other primary metabolites are desirable, since promoting additional water stress on wounded plant tissue could alter the wound-induced activation of primary and secondary metabolic pathways.

The present research work objective was to evaluate the separate and combined effects of water and wounding stress on the primary and secondary metabolism of carrots, which was evaluated at the gene expression level and on the accumulation of primary (shikimic acid and L-phe) and secondary metabolites (phenolic compounds and lignin). The information presented herein is of major relevance for the pharmaceutical, dietary supplements and food industries, since it gives insights on how the application of additional water stress in wounded carrots could alter the accumulation of bioactive plant metabolites with high commercial value.

## Materials and methods

### Chemicals

Shikimic acid, CHA, FA, *p*-CA, PCA, gallic acid (GA), *t*-cinnamic acid (*t*-CA), methanol (HPLC grade), water (HPLC grade), and orthophosphoric acid, were purchased from Sigma Chemical Co. (St. Louis, MO, USA). Monopotassium phosphate (KH_2_PO_4_), hydrochloric acid (HCl), sodium hydroxide (NaOH), and sulfuric acid (H_2_SO_4_) were purchased from Desarrollo de Especialidades Químicas S.A. de C.V. (San Nicolas de los Garza, NL, Mexico).

### Plant material, processing, and storage studies

**Figure 1:**
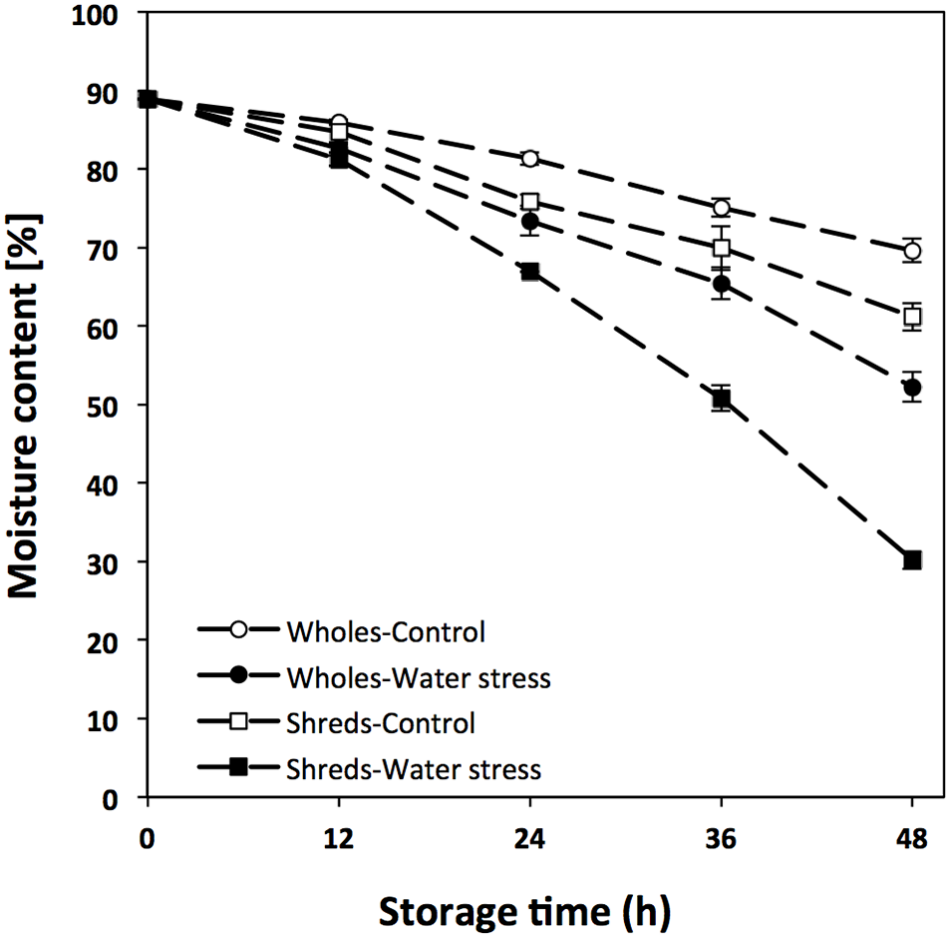
**Moisture content [%] of whole and shredded carrots stored for 48 h under control and water stress conditions**. Values represent the mean of 3 replicates with their standard error bars.

Carrots (*Daucus carota*) were obtained from a local grocery store (HEB, Monterrey, NL, Mexico), sorted, washed and disinfected with chlorinated water (200 ppm, pH 6.5). Shredded carrots were obtained by wounding whole carrots with a vegetable shredder (diameter of 0.7 cm). Wholes and wounded carrots (500 g) were placed in open plastic containers with capacity of 5.7 L (Sterilite, Townsend, MA, USA) and stored for 48 h under two different incubation conditions: induced water stress and control conditions. Induced water stress conditions were achieved by placing carrots (wholes and shreds) inside of a drying oven (EDEL Ingenieros, Monterrey, NL, Mexico) with forced air convection operating at 25°C with ~20% of relative humidity. Samples used as a control were stored at 25°C in an incubator (VWR, Radnor, PA, USA) with ~35% of relative humidity and no forced air convection. These conditions allowed the generation of samples with different percentages of moisture content during storage (Figure 1), which were used for the chemical and gene expression analyses described herein.

Samples were collected every 12 h during the storage period (48 h). Variables such as moisture content and the expression of genes related with primary and secondary metabolism of carrots were evaluated before and during storage of the different treatments. Likewise, the concentration of individual phenolic compounds, shikimic acid, L-phenylalanine (L-phe), and total lignin were determined. Gene expression analyses were performed in fresh tissue, whereas all other chemical analyses were determined in freeze-dried carrots obtained with a freeze-drying system (Labconco Corp, Kansas City, Missouri, MO, USA).

### Moisture (%) and water loss determinations

Moisture (%) of time 0 h samples was determined by the air-oven method (AACC 44-15A), whereas moisture content (%) of stored samples was calculated considering water loss of samples using Equation (1).

$$\begin{array}{l} \%\;Moisture\;at\;t_{xh}=\%\;Moisture\;at\;t_{0h}-\left[\frac{wt_{0h}-wt_{xh}}{wt_{0h}}\times 100\right] \end{array}$$

Where *x* is the sampling time (12, 24, 36, and 48 h) and *wt* is the sample weight.

### Identification and quantification of shikimic acid

For the extraction of shikimic acid, freeze-dried carrot (0.05 g) was added with methanol (2 mL), sonicated for 5 min, and vortexed for 30 s. The homogenates were stored overnight in total darkness (~12 h at 4°C) and then centrifuged (10,000 × *g*, 15 min, 4°C). The clear supernatant was microfiltered using nylon membranes (0.45 μm, VWR, Radnor, PA, USA) prior to injection to the chromatographic system.

Shikimic acid analyses were performed by HPLC-PDA according to a method previously reported (Avula et al., 2009) with slight modification. The methanol extracts (10 μL) were injected in the HPLC system, which was composed of a quaternary pump, an autosampler, and a diode array detector (1260 Infinity, Agilent Technologies, Santa Clara, CA, USA). Shikimic acid was separated on a 4.6 × 250 mm, 5 μm, NH_2_ column (Luna, Phenomenex, Torrance, CA, USA) with a C18 ward column and maintained at 30°C. The mobile phases consisted of 10 mM KH_2_PO_4_ adjusted to pH 4.8 with orthophosphoric acid (phase A) and methanol (phase B). The gradient solvent system was 0/50, 12/50, 20/0, 30/0, and 45/50 (min/% phase A) at a constant flow rate of 1 mL/min. Chromatographic data was processed with the OpenLAB CDS ChemStation software (Agilent Technologies, Santa Clara, CA, USA).

The detection wavelength was at 210 nm and shikimic acid was identified by comparison of the retention time and PDA spectra with an authentic standard. For the quantification of shikimic acid, a standard curve of the compound was prepared at a range of 5–250 mg/L (ppm). Shikimic acid concentration was expressed as mg of shikimic acid per kg of carrots dry weight (DW).

### Identification and quantification of individual phenolic compounds

For the extraction of individual phenolic compounds, freeze-dried carrot (0.5 g) was vortexed with methanol (20 mL) for 30 s. The homogenates were stored overnight in total darkness (~12 h at 4°C) and then centrifuged (10,000 × *g*, 15 min, 4°C). The clear supernatant was microfiltered using nylon membranes (0.45 μm, VWR, Radnor, PA, USA) prior to injection to the chromatographic system.

Individual phenolic compounds were identified and quantified according to a method previously described (Torres-Contreras et al., 2014a). The identification of individual phenolic compounds was based on their PDA spectra and ESI-MS fragmentation patterns as compared with authentic standards and previous reports (Clifford et al., 2003; Jacobo-Velázquez et al., 2011; Becerra-Moreno et al., 2012; Torres-Contreras et al., 2014a,b). For the quantification of individual phenolic compounds, standard curves of PCA, GA, CHA, *p*-CA and FA were prepared at a range of 5–250 mg/L (ppm). The concentration of the phytochemicals was expressed as mg of each individual compound per kg of carrots DW. Likewise, the sum of all individual compounds identified was expressed as total phenolic compounds.

### Identification and quantification L-phenylalanine (L-phe)

Quantification of L-phe was achieved by determining the complete free amino acids (*f* -AA) profile in carrots. Derivatization and analysis of *f* -AA were performed according to the Instruction Manual of the Waters AccQ•Tag Chemistry Package with slight modifications for *f* -AA. Freeze-dried carrots (0.05 g) were vortexed with 20 mM HCl (4.3 mL, 30 s). Subsequently, the homogenates were incubated on a rotary shaker (60 rpm, 30 min, 25°C) and centrifuged (10,000 × *g*, 10 min, 4°C). The clear supernatant was subjected to microfiltration using nylon membranes (0.45 μm, VWR, Radnor, PA, USA) prior to derivatization and injection to the chromatographic system.

The *f* -AA extracts were derivatized with the Waters AccQ•Fluor Reagent Kit and analyses were performed by HPLC-FLD according to the method reported by the Instruction Manual. The derivatized *f* -AA sample (10 μL) was injected in the HPLC system, which was composed of two binary pumps, an autosampler, and a fluorescence detector (1200 Infinity, Agilent Technologies, Santa Clara, CA, USA). Compounds (*f* -AA) were separated on a 3.9 × 150 mm, 4 μm, C18 reverse phase Waters AccQ•Tag column (Nova-Pak, Milford, MA, USA). The mobile phases, gradient solvent system and the external calibration standard were according to the Instruction Manual.

Chromatographic data was processed with the OpenLAB CDS ChemStation software. The excitation and emission wavelengths were 250 and 395 nm, respectively and the concentration was expressed as mg of L-phe per kg of carrots DW.

### Total lignin content determination

Total lignin content was determined by the Klason lignin procedure reported by Templeton and Ehrman (1995). Briefly, freeze-dried carrot tissue (1 g) was place in a 20 × 150 mm Pyrex glass tube. Then, 72% sulfuric acid at 4°C (15 mL) was added and mixed with a glass rod until the sample was completely wet. The sample was hydrolyzed for 2 h at room temperature (~20°C) and stirred every 15 min to ensure a homogeneous mixture. The hydrolyzed solutions were transferred to an Erlenmeyer flask. Then 560 mL of distilled water were added, and placed under heating (300°C) and reflux (5°C) for 4 h. Finally, the hydrolyzed solution was filtered under vacuum through a gooch filter. Soluble lignin was determined taking an aliquot (1 mL) of the filtrate and measuring absorbance at 240 nm. For the determination of insoluble lignin, hot water was used to remove quantitatively the content in the Erlenmeyer flask through the gooch filter and wash the residue under vacuum filtration to eliminate all acid. Then the gooch filter and its content were placed overnight in a drying oven (VWR, Radnor, PA, USA) at 105°C. The filter was cooled down in a desiccator and weighed to measure the corresponding weight of the filter, ashes and insoluble acid lignin. After being weighed, the filter was placed in a muffle (Thermo Scientific, Waltham, MA, USA) at 575°C for 3 h. Finally the filter was cooled down in a desiccator and weighed to measure the corresponding weight of the gooch filter and the acid ashes. Total lignin was determined by adding the values of the soluble and insoluble lignin and concentration was expressed as mg per kg DW.

### Gene expression analysis

Total RNA from samples before and during storage was extracted following the hot borate method (Wan and Wilkins, 1994). RNA quality (260/280 ratio) and quantity (260 nm) were determined using a NanoDrop 1000 spectrophotometer (Thermo Scientific, Waltham, MA, USA). Likewise, RNA integrity was determined on 1% (w/v) agarose gels. Total RNA was treated with DNAse using the RNase-Free DNase Set (Qiagen, Hilden, NRW, Germany) and cleaned up using the RNeasy Plant Mini Kit (Qiagen, Hilden, NRW, Germany) following the manufacture's recommendations. First strand of cDNA was obtained by reverse transcription reaction using AffinityScript QPCR cDNA Synthesis Kit (Agilent Technologies, Santa Clara, CA, USA) with random primers according to standard procedures. The single stranded cDNA obtained was subjected to qRT-PCR in a Rotor-Gene 3000 system (Corbett Life Science, San Francisco, CA, USA) with a 36-well rotor using Brilliant III Ultra-Fast SYBR Green QPCR Master Mix (Agilent Technologies, Santa Clara, CA, USA) and gene-specific primers (Quimera Biolabs, Ensenada, BC, Mexico) designed as previously reported (Jacobo-Velázquez et al., 2015) and listed in Table S1 (Supplementary Material). qRT-PCR conditions, procedure and analyses were performed as described by Salzman et al. (2005). Experiments were performed by triplicate and the expression values were normalized against α-tubulin, because it has been demonstrated that it behaves as housekeeping gene when analyzing gene expression of carrots treated with wounding stress (Jacobo-Velázquez et al., 2015). Amplification specificity was determined by dissociation curve analysis, and the amplification product sizes were confirmed in an agarose gel to ensure the absence of non-specific PCR products.

The relative expressions of the genes were calculated as 2^∧^(ΔΔCt), where:

$$\begin{array}{lll} \Delta \Delta \text{Ct} & = & \left(\Delta {\text{Ct}}_{\;0\text{ h samples cDNA}}\right)\;-\;\left(\Delta {\text{Ct}}_{\text{ Sample cDNA}}\right)\\ \Delta \text{Ct} & = & \left(\text{mean Ct cDN}{\text{A}}_{\text{Test primers}}\right)\\ -\left(\text{mean Ct cDNA}{\;}_{\text{α}-\text{Tubulin primers}}\right) \end{array}$$

### Statistical analysis

Replication was achieved by repeating treatment under the same conditions. The control and water stress conditions were run concurrently. All reported data were pooled from repeated independent treatments. There were three replicates per treatment (*n* = 3). Analyses of variance (ANOVA) were conducted to determine main effects and interactions, using JMP software version 5.0 (SAS Institute Inc. Cary, NC, USA) and mean separations performed using LSD test (*p* < 0.05) for primary and secondary metabolites for biological comparisons and technological applications. *t*-Student test comparison was performed to determine significant difference between data for relative expression of genes.

## Results

### Effect of water and wounding stress on moisture content of carrots

The moisture content of carrots stored under water stress and control conditions is shown in Figure 1. As expected, the application of water stress significantly decreased the moisture content of wholes and shredded carrots during storage, where wounded tissue showed a significant higher moisture loss. The moisture content of time 0 h samples was ~89%, whereas wholes stored for 48 h presented moisture contents of ~52 and ~70%, for samples stored under waters stress and control conditions, respectively. Likewise, the moisture content of wounded carrots treated with water stress decreased to ~30% at 48 h of storage, whereas wounded carrots stored under control conditions showed a moisture content of ~61%. These samples with different degrees of water loss were used for the analysis of shikimic acid, L-phe, phenolic compounds, and lignin. In addition, the analysis of genes related with the biosynthesis of primary (shikimic acid and aromatic amino acids) and secondary (phenolic compounds and lignin) metabolites were evaluated in order to better understand the effect of wounding and water stress on the activation of primary and secondary metabolic pathways in plants.

### Effect of wounding and water stress on the expression of primary metabolism related genes

The relative expression of genes with putative function identified as 3-deoxy-D-arabino-heptulosanate synthase (DAHP synthase), 5-enolpyrovylshikimate 3-phosphate synthase (EPSP synthase), and chorismate mutase-prephenate dehydratase (CMPD), which are related with the biosynthesis of shikimic acid and L-phe, was evaluated during storage of all treatments (Table 1). The relative expression of the three genes was significantly affected (*p* < 0.001) by all factors evaluated (wounding stress, water stress and storage time) (Table 1). Wholes treated with water stress showed higher relative expression of the three genes at 48 h of storage as compared with their controls, indicating that water stress alone has a significant impact on the activation of primary metabolism related genes. Likewise, carrot tissue treated with wounding stress showed higher relative expression of DAHP synthase, EPSP synthase, and CMPD, whereas their wound-induced activation was highly promoted when additional water stress was applied, showing the highest relative expression at 24 h of storage. Thereafter, the expression of DAHP synthase, EPSP synthase, and CMPD in the wounded tissue started to decrease.

**Table 1:** **Relative expression of genes with putative function related with the primary metabolism in whole and shredded carrots stored for 48 h under control and water stress conditions**.

**Sample**	**Storage time**	**Treatment**	**Relative expression^a^^,^^b^**		
			**DAHP synthase**	**EPSP synthase**	**CMPD**
Wholes	12 h	Control	0.70 ± 0.02^***^	1.06 ± 0.07**	13.76 ± 0.84^***^
		Water stress	0.44 ± 0.00	0.69 ± 0.01	3.86 ± 0.04
	24 h	Control	0.42 ± 0.01	0.86 ± 0.02	21.29 ± 0.53^***^
		Water stress	0.88 ± 0.02^***^	1.89 ± 0.12^**^	0.18 ± 0.01
	36 h	Control	0.97 ± 0.03	3.01 ± 0.21^**^	16.06 ± 0.67
		Water stress	0.98 ± 0.00	1.66 ± 0.09	19.68 ± 1.44
	48 h	Control	0.93 ± 0.01	2.96 ± 0.05	38.52 ± 1.42
		Water stress	3.58 ± 0.08^***^	10.55 ± 0.72^***^	76.90 ± 3.31^***^
Shreds	12 h	Control	115.52 ± 48.35	40.73 ± 9.55	123.97 ± 59.51
		Water stress	2030.92 ± 146.67^***^	1094.03 ± 81.32^***^	2692.74 ± 221.34^***^
	24 h	Control	220.34 ± 21.80	135.96 ± 9.64	646.67 ± 48.85
		Water stress	4538.45 ± 522.50^***^	1152.40 ± 49.90^***^	5251.89 ± 646.77^***^
	36 h	Control	76.16 ± 4.71	31.94 ± 2.81	135.18 ± 15.90
		Water stress	252.77 ± 8.64^***^	138.69 ± 8.82^***^	687.88 ± 29.89^***^
	48 h	Control	85.56 ± 2.35	84.59 ± 4.22^**^	273.62 ± 16.16
		Water stress	96.92 ± 2.40^*^	50.03 ± 2.48	505.05 ± 21.18^***^
**SIGNIFICANCE^c^**					
Wounding stress			^***^	^***^	^***^
Water stress			^***^	^***^	^***^
Storage time			^***^	^***^	^***^
Wounding stress x water stress			^***^	^***^	^***^
Water stress x storage time			^***^	^***^	^***^
Wounding stress x storage time			^***^	^***^	^***^
Wounding stress x water stress x storage time			^***^	^***^	^***^

a *Data represents the mean of 3 replicates ± standard error of the mean*.

b Values with an asterisk indicate significant difference between the control and water stress treated samples by analysis of variance (ANOVA) ^*^p < 0.05, ^**^p < 0.01,^***^p < 0.001.

c Asterisks indicate that main effects and interactions are significantly different by ANOVA. NS, non significant; ^*^p < 0.05; ^**^p < 0.01;^***^p < 0.001. DAHP synthase, 3-deoxy-D-arabino-heptulosanate synthase; EPSP synthase, 5-enolpyrovylshikimate 3-phosphate synthase; CMPD, Chorismate mutase-prephenate dehydratase.

### Effect of wounding and water stress on the accumulation of primary metabolites

The concentration of shikimic acid and L-phe during storage of whole and shredded carrots treated and non-treated with water stress is shown in Table 2. Both primary metabolites are needed as carbon sources for the biosynthesis of phenolic compounds and other secondary metabolites. Shikimic acid content was significantly affected (*p* < 0.001) by wounding stress and storage time. However, water stress alone did not affect shikimic acid content. All interactions between the factors evaluated (wounding stress, water stress, and storage time) significantly influenced (*p* < 0.01) shikimic acid content in samples. Compared with shikimic acid levels in time 0 h samples (98 ± 12.0 mg/kg), the highest accumulation of shikimic acid in wholes was observed at 12 h of storage in water stress treated samples (208.1 ± 16.8 mg/kg), whereas for shreds the highest levels were detected at 24 h of storage of the controls (234.7 ± 20.6 mg/kg). Thereafter, shikimic acid content decreased to levels even lower than those quantified in time 0 h samples.

**Table 2:** **Effects of wounding and water stress on the concentration of shikimic acid and phenylalanine during storage of carrots**.

**Samples**	**Storage time (h)**	**Treatment**	**Shikimic acid concentration (mg/kg DW)^i^^,^^ii^**	**L-Phenylalanine concentration (mg/kg DW) ^i^^,^^ii^**
Wholes	0	Control	98.4±12.0^d^	343.5±17.8^e, f^
	12	Control	160.8±9.5^b^	423.1±20.1^d^
		Water stress	208.1±16.8^a^	366.7±6.1^e^
	24	Control	33.1±3.5^g, h, i^	510.6±17.1^c^
		Water stress	57.2±1.2^e, f, g, h^	302.5±17.2^f^
	36	Control	30.6±5.0^h, i^	621.9±30.5^b^
		Water stress	48.4±1.6^f, g, h^	418.2±14.6^d^
	48	Control	20.1±0.5^i^	666.1±20.5^a^
		Water stress	19.4±0.6^i^	468.5±25.0^c^
Shreds	12	Control	140.7±7.8^b, c^	250.3±10.5^g^
		Water stress	93.3±6.9^d^	220.9±13.9^g^
	24	Control	234.7±20.6^a^	100.4±2.2^h, i^
		Water stress	133.3±12.6^c^	134.5±9.9^h^
	36	Control	63.1±7.7^e, f^	78.3±5.2^i^
		Water stress	82.5±13.7^d, e^	79.1±4.1^i^
	48	Control	47.8±1.0^f, g, h^	68.2±1.1^i^
		Water stress	58.9±2.3^e, f, g^	79.5±3.1^i^
**SIGNIFICANCE^iii^**				
Wounding stress			^***^	^***^
Water stress			NS	^***^
Storage time			^***^	^***^
Wounding stress x water stress			^***^	^***^
Water stress x storage time			^**^	^***^
Wounding stress x storage time			^***^	^***^
Wounding stress x water stress x storage time			^***^	^***^

i *Data represents the mean of 3 replicates ± standard error of the mean*.

ii *Different letters in the same column indicate statistical difference by the LSD test (p < 0.05)*.

iii *Asterisks indicate that main effects and interactions are significantly different by analyses of variance (ANOVA)*.

NS, non significant; ^*^p < 0.05;

** *p < 0.01*;

*** *p < 0.001*.

L-phe content was significantly affected (*p* < 0.001) by all factors evaluated (Table 2). For wholes control, L-phe content increased through time showing the highest levels at 48 h of storage, whereas for wholes treated with water stress L-phe content showed a significant decrease after 12 h. For wounded carrots, L-phe content decreased during storage for both the control and water stress treated samples, and non-significant effect (*p* > 0.05) of water stress was observed.

### Effect of wounding and water stress on the expression of secondary metabolism related genes

The relative expression of genes with putative function identified as phenylalanine ammonia-lyase (PAL), *trans*-cinnamate 4-monooxygenase (C4H), 4-coumarate-CoA ligase (4CL), caffeoyl-CoA 3-*O*-methyltransferase (CCoAOMT), cinnamoyl-CoA reductase (CCR), and cinnamyl alcohol dehydrogenase (CAD) was evaluated during 48 h of storage in all treatments (Table 3). PAL, C4H, and 4CL genes are related with the biosynthesis of hydroxycinnamic acids, whereas CCoAOMT, CCR, and CAD are involved on the conversion of hydroxycinnamic acids into lignin. Samples treated with wounding stress showed higher relative expression of all genes involved in the biosynthesis of phenolics and lignin, as compared with wholes, particularly when water stress was applied. Likewise, as observed for genes related with the primary metabolism, at 48 h of storage whole carrots treated with water stress presented higher relative expression of secondary metabolism related genes, indicating that water stress alone has a significant effect on the activation of these six genes. For wholes treated with water stress, their highest relative expression was observed at 48 h of storage. On the other hand, carrots treated with wounding stress alone showed the highest relative expression of PAL, C4H, 4CL, CCoAOMT, CCR, and CAD at 24 h of storage, whereas an earlier activation of PAL, 4CL, CCoAOMT, and CAD was observed when shredded carrots were treated with water stress. In wounded tissue, once the highest relative expression of genes related with the phenylpropanoid metabolism was achieved it started to decrease.

**Table 3:** **Relative expression of genes with putative function related with the secondary metabolism in whole and shredded carrots stored for 48 h under control and water stress conditions**.

**Sample**	**Storage time (h)**	**Treatment**	**Relative expression^a^^,^^b^**					
			**PAL**	**C4H**	**4CL**	**CCoAOMT**	**CCR**	**CAD**
Wholes	12	Control	3.58 ± 0.24	0.82 ± 0.06	7.14 ± 0.38^*^	3.11 ± 0.21^*^	5.79 ± 0.28	0.07 ± 0.00
		Water stress	3.33 ± 0.04	1.33 ± 0.01^***^	5.88 ± 0.11	2.34 ± 0.04	7.40 ± 0.02^**^	0.11 ± 0.01^**^
	24	Control	4.30 ± 0.10	0.72 ± 0.00	13.58 ± 0.10	1.72 ± 0.03	4.64 ± 0.01	0.65 ± 0.01
		Water stress	13.80 ± 0.39^***^	3.94 ± 0.12^***^	18.30 ± 0.83^**^	4.68 ± 0.31^***^	5.95 ± 0.15^**^	1.55 ± 0.01^***^
	36	Control	5.78 ± 0.09^**^	1.70 ± 0.03	12.62 ± 0.46^**^	4.50 ± 0.21	15.39 ± 0.19^***^	2.04 ± 0.02^***^
		Water stress	4.73 ± 0.18	2.39 ± 0.09^**^	8.48 ± 0.43	4.49 ± 0.22	4.91 ± 0.05	1.04 ± 0.02
	48	Control	6.05 ± 0.14	2.04 ± 0.05	20.24 ± 1.00	3.76 ± 0.12	6.80 ± 0.06	2.31 ± 0.02
		Water stress	43.38 ± 0.39^***^	13.64 ± 0.35^***^	209.32 ± 9.51^***^	26.85 ± 1.07^***^	108.03 ± 2.59^***^	4.33 ± 0.03^***^
Shreds	12	Control	253.77 ± 77.10	148.66 ± 60.17	4017.67 ± 108.41	275.62 ± 40.90	6506.38 ± 249.67	30.64 ± 10.73
		Water stress	3662.88 ± 272.25^***^	1661.69 ± 97.63^***^	121367.55 ± 8471.93^***^	5723.02 ± 468.37^***^	76585.85 ± 5361.12^***^	533.62 ± 68.85^***^
	24	Control	585.90 ± 187.51	272.69 ± 45.94	9682.91 ± 724.84	622.51 ± 120.26	11928.54 ± 570.46	21.08 ± 1.53
		Water stress	2797.24 ± 236.92^***^	1958.33 ± 104.95^***^	81974.20 ± 3652.39^***^	4187.06 ± 217.29^***^	92083.54 ± 4155.36^***^	227.20 ± 41.33^***^
	36	Control	194.49 ± 11.23	97.68 ± 4.74	2655.41 ± 227.99	146.37 ± 11.79	6082.60 ± 467.58	10.36 ± 0.28
		Water stress	367.78 ± 15.98^***^	359.32 ± 4.64^***^	10235.38 ± 419.89^***^	531.61 ± 19.89^***^	15916.72 ± 291.07^***^	43.62 ± 3.20^***^
	48	Control	351.22 ± 12.41^***^	138.23 ± 3.54^**^	6531.01 ± 305.19^***^	144.49 ± 9.41	9329.33 ± 233.16^***^	11.11 ± 0.30^**^
		Water stress	140.90 ± 3.15	115.10 ± 1.07	3067.82 ± 165.28	120.39 ± 7.14	4138.47 ± 179.00	9.30 ± 0.05
**SIGNIFICANCE^c^**								
Wounding stress			^***^	^***^	^***^	^***^	^***^	^***^
Water stress			^***^	^***^	^***^	^***^	^***^	^***^
Storage time			^***^	^***^	^***^	^***^	^***^	^***^
Wounding stress x water stress			^***^	^***^	^***^	^***^	^***^	^***^
Water stress x storage time			^***^	^***^	^***^	^***^	^***^	^***^
Wounding stress x storage time			^***^	^***^	^***^	^***^	^***^	^***^
Wounding stress x water stress x storage time			^***^	^***^	^***^	^***^	^***^	^***^

a *Data represents the mean of 3 replicates ± standard error of the mean*.

b Values with an asterisk indicate significant difference between the control and water stress treated samples by analysis of variance (ANOVA) *p < 0.05, **p < 0.01,^***^p < 0.001.

c Asterisks indicate that main effects and interactions are significantly different by ANOVA. NS, non significant, *p < 0.05, **p < 0.01, ^***^p < 0.001. PAL, Phenylalanine ammonia-lyase; C4H, trans-cinnamate 4-monooxygenase; 4CL, 4-coumarate-coa ligase; CCoAOMT, Caffeoyl-CoA 3-O-methyltransferase; CCR, Cinnamoyl-CoA reductase; CAD, Cinnamyl alcohol dehydrogenase.

### Effect of water and wounding stress on the accumulation of total phenolic compounds and lignin

**Figure 2:**
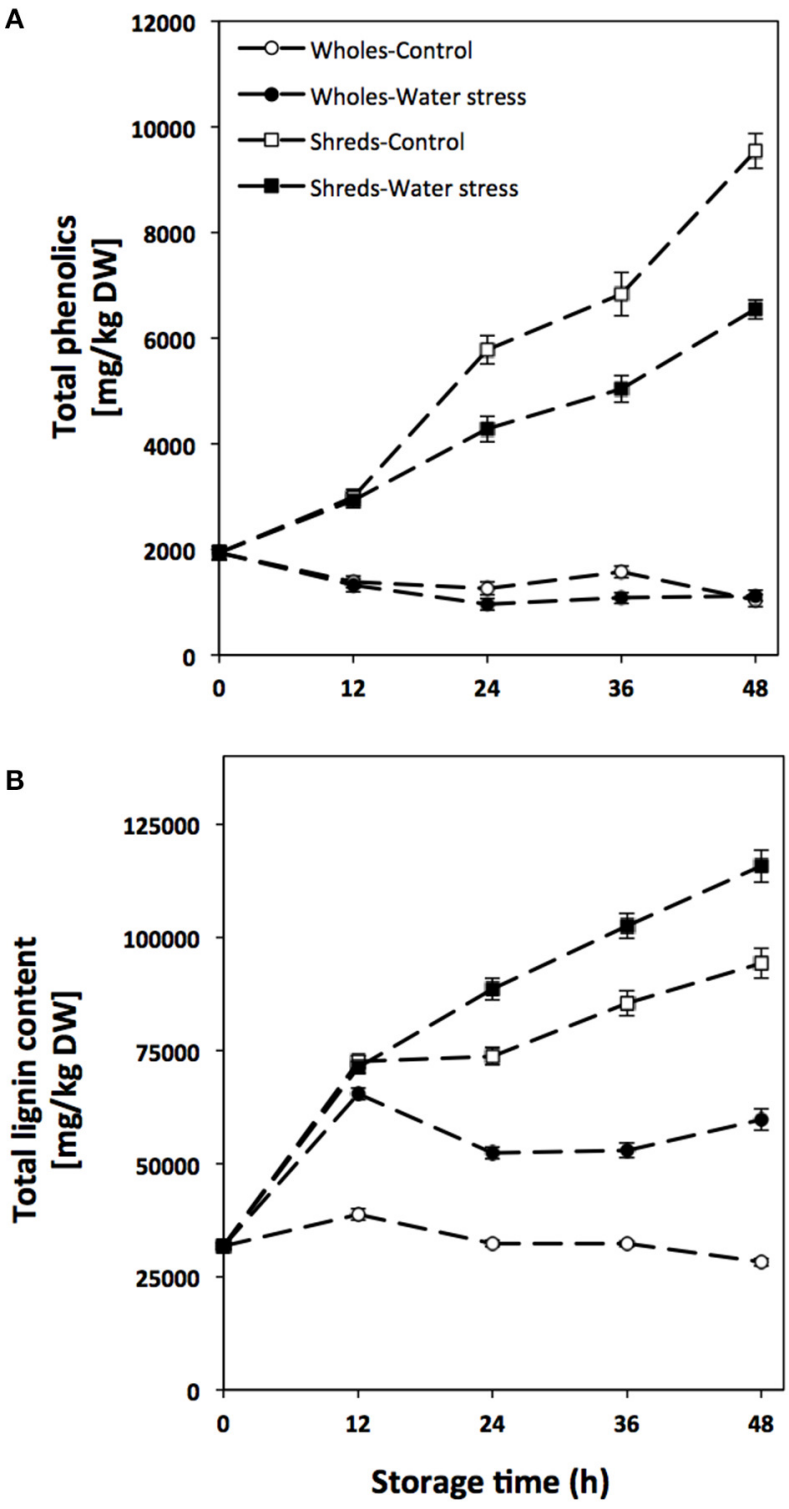
**Total phenolics (A) and total lignin content (B) of whole and shredded carrots stored for 48 h under control and water stress conditions**. Values represent the mean of 3 replicates with their standard error bars.

The concentration of total phenolics and lignin during storage of carrots treated and non-treated with wounding and water stress is shown in Figure 2. Whole carrots showed a decrease in total phenolic content during storage, presenting lower levels those treated with water stress (Figure 2A). On the other hand, wounded carrots showed a gradual increase on the concentration of total phenols throughout storage time. However, the wound-induced accumulation of total phenols was decreased when the tissue was stored under water stress conditions. For instance, at 48 h of storage, shredded carrots showed 360% higher levels of total phenolics as compared with time 0 h, whereas the increase in water stress treated shreds was of 220%.

Regarding total lignin, the concentration in carrots was also significantly affected (*p* < 0.001) by all factors evaluated as well as by their interactions (see Table S2). For the control and water stress treated whole carrots, total lignin content increased through time showing the highest levels at 12 h of storage and thereafter the content started to decrease. For shreds, total lignin levels gradually increased during storage where carrots treated with wounding in combination with water stress showed 23% higher accumulation of total lignin than the samples treated with wounding alone.

### Effect of water and wounding stress on the phenolic profiles

To better elucidate the effect of wounding and water stress on the phenylpropanoid metabolism of carrots it was considered relevant to evaluate the changes in their phenolic profiles during storage of the stressed tissue. The identification of individual phenolic compounds in carrots included protocatechuic acid (PCA), gallic acid derivative (GAD), CHA, 3,5-diCQA, 3-hydroxy dihydro chlorogenic acid (3-*h*CQA), 4,5-diCQA, *p*-coumaric acid (*p*-CA), ferulic acid (FA), *p*-CA derivative (*p*-CAD), ferulic acid derivative (FAD), and isocoumarin (IC) (see Figure S1 and Table S3).

3,5-diCQA and *p*-CAD were not identified in whole carrots before storage (time 0 h samples), and both compounds remained undetected during storage time (Table 4). Likewise, 4,5-diCQA was not present in time 0 h samples, however this compound was accumulated in wholes treated and non-treated with water stress, achieving higher concentrations under control storage conditions. Likewise, the concentration of GAD slightly increased during storage of whole carrots, where no significant difference (*p* > 0.05) was observed between the control and water stress treated samples. While FAD content increased during the first 24 h of storage of whole carrots stored under control conditions, wholes treated with water stress showed a gradual decrease in FAD. CHA, the main phenolic compound identified in time 0 h samples, dramatically decreased in concentration during storage of whole carrots, showing lower levels those samples stored under water stress conditions. 3-*h*CQA content also decreased during storage of whole carrots, particularly under control conditions. Likewise, during storage of whole carrots under control conditions, PCA, *p*-CA, FA, and IC contents remained at similar concentration as compared with whole carrots before storage (control 0 h samples). On the other hand, water stress induced a significant accumulation of PCA and a significant decrement in FA content throughout storage time of whole carrots (Table 4).

**Table 4:** **Effects of wounding and water stress on the concentration of individual phenolic compounds during storage of carrots**.

**Sample**	**Storage time (h)**	**Treatment**	**Individual phenolic compounds concentration (mg/kg DW)^i^^,^^ii^^,^^iii^^,^^iv^^,^**					
			**PCA**		**GAD**	**CHA**	**3,5-diCQA**	**3-*h*CQA**
Wholes	0	Control	69.4±1.0^f, g, h^		91.1±1.4^c^	1047.0±32.2^g^	ND	398.3±15.7^b^
	12	Control	58.9±0.6^k^		98.2±1.1^b^	591.0±15.1^h, i^	ND	88.2±0.3^i, j^
		Water stress	73.3±0.5^e, f^		97.1±1.3^b^	532.5±26.6^h, i, j^	ND	132.7±3.7^h^
	24	Control	59.6±0.5^k^		82.1±1.1^d^	468.1±18.4^h, i, j^	ND	66.4±1.2^j^
		Water stress	71.3±0.8^e, f, g^		70.0±0.4^e^	256.9±14.0^i, j^	ND	91.8±4.2^i^
	36	Control	63.1±0.5^j, k^		98.4±2.0^b^	758.5±12.9^g, h^	ND	72.3±0.7^i, j^
		Water stress	65.9±1.2^h, i, j^		96.8±0.9^b^	236.6±3.8^i, j^	ND	193.5±2.5^g^
	48	Control	65.1±0.4^h, i, j^		106.7±2.1^a^	267.1±9.1^i, j^	ND	65.1±1.2^j^
		Water stress	74.1±0.9^d, e, f^		107.4±2.9^a^	154.7±5.8^j^	ND	258.2±3.6^f^
Shreds	12	Control	63.6±1.1^i, j, k^		57.4±1.7^g, h^	1919.4±50.0^f^	66.4±0.8^e^	257.7±6.2^f^
		Water stress	68.0±0.8^g, h, i^		64.0±0.9^f^	1763.0±41.7^f^	71.3±1.2^c^	321.4±11.4^d^
	24	Control	74.3±0.9^d, e^		59.4±0.8^g^	4322.8±168.3^c^	68.5±0.3^d, e^	282.4±5.5^e^
		Water stress	103.2±2.3^b^		65.0±1.4^f^	2769.4±140.3^e^	74.6±1.0^b^	349.9±11.8^c^
	36	Control	78.2±1.1^d^		57.5±1.4^g, h^	4940.1±308.2^b^	69.6±1.1^c, d^	315.3±7.1^d^
		Water stress	123.8±2.6^a^		54.2±0.7^h, i^	3219.7±148.6^d^	76.5±1.7^b^	349.2±16.2^c^
	48	Control	83.0±2.7^c^		51.4±1.6^i^	6344.2±318.6^a^	69.8±2.1^c, d^	348.3±13.2^c^
		Water stress	124.2±4.3^a^		52.6±0.8^i^	4260.1±189.9^c^	83.8±1.3^a^	452.2±7.2^a^
**SIGNIFICANCE^v^**								
Wounding stress			^***^		^***^	^***^	^***^	^***^
Water stress			^***^		NS	^***^	^***^	^***^
Storage time			^***^		^***^	^***^	^***^	^***^
Wounding stress x water stress			^***^		^***^	^***^	^***^	^*^
Water stress x storage time			^***^		^*^	^***^	^***^	^***^
Wounding stress x storage time			^***^		^***^	^***^	^***^	^***^
Wounding stress x water stress x storage time			^***^		^***^	^***^	^***^	^***^
**Sample**	**Storage time (h)**	**Treatment**	**Individual phenolic compounds concentration (mg/kg DW) ^i^^,^^ii^^,^^iii^^,^^iv^^,^**					
			**4,5-diCQA**	***p*****-CA**	**FA**	***p*****-CAD**	**FAD**	**IC**
Wholes	0	Control	ND	102.4±1.7^a, b^	116.5±2.1^c, d, e^	ND	131.5±2.3^f^	147.4±8.8^e^
	12	Control	86.2±0.9^a, b^	98.3±0.9^d, e, f^	111.9±1.1^f, g, h^	ND	149.0±3.2^d, e^	85.6±2.6^e^
		Water stress	73.2±1.2^d, e^	95.9±0.4^f, g^	110.9±0.8^g, h, i^	ND	125.1±1.8^f^	84.4±1.4^e^
	24	Control	69.4±1.8^e, f, g^	96.5±1.3^e, f, g^	113.0±1.4^e, f, g^	ND	170.7±0.9^a, b^	100.9±4.1^e^
		Water stress	68.0±1.1^g^	94.7±0.8^g^	108.4±1.3^h, i, j^	ND	109.6±0.8^g^	75.3±2.5^e^
	36	Control	74.4±0.9^c, d^	96.1±0.9^f, g^	115.5±1.3^d, e, f^	ND	152.4±2.1^d, e^	115.1±2.2^e^
		Water stress	68.6±1.3^f, g^	103.4±1.5^a, b^	106.9±1.8^i, j^	ND	106.2±1.0^g^	75.5±2.1^e^
	48	Control	77.8±0.5^*c*^	99.0±0.4^c, d, e^	119.5±1.3^c, d^	ND	127.2±1.1^f^	71.6±2.2^*e*^
		Water stress	67.6±1.3^g^	102.0±0.6^a, b^	105.1±1.5^j^	ND	105.8±1.5^g^	99.4±2.6^e^
Shreds	12	Control	74.6±0.8^c, d^	ND	120.6±0.7^c^	119.8±1.2^f^	169.6±2.7^a, b^	113.3±1.7^e^
		Water stress	88.2±1.1^a^	97.8±1.6^e, f^	112.0±2.0^f, g, h^	137.4±3.0^d^	133.2±3.3^f^	85.5±2.6^e^
	24	Control	68.1±0.3^g^	ND	128.3±1.0^b^	123.4±0.7^e, f^	174.6±7.2^a^	425.6±8.4^d^
		Water stress	87.7±2.1^a, b^	101.3±1.1^a, b, c^	117.9±1.3^c, d^	141.7±2.5^d^	128.0±3.9^f^	357.2±18.3^d^
	36	Control	62.3±1.0^h^	ND	129.2±2.3^b^	126.2±2.1^e^	144.6±0.9^e^	870.1±21.0^b^
		Water stress	83.9±2.0^b^	103.4±1.1^a^	119.0±0.5^c, d^	147.2±2.5^c^	131.8±2.0^f^	640.2±20.9^c^
	48	Control	59.2±1.0^h^	ND	134.7±1.7^a^	195.4±2.3^b^	156.5±3.6^c, d^	2046.2±95.5^a^
		Water stress	72.3±2.7^d, e, f^	100.7±1.0^b, c, d^	120.3±1.3^c^	218.8±3.4^a^	164.0±1.7^b, c^	892.1±36.4^b^
**SIGNIFICANCE^v^**								
Wounding stress			^*^	^***^	^***^	^***^	^***^	^***^
Water stress			^***^	^***^	^***^	^***^	^***^	^***^
Storage time			^***^	^***^	^***^	^***^	^***^	^***^
Wounding stress x water stress			^***^	^***^	^*^	^***^	^***^	^***^
Water stress x storage time			^***^	^***^	^***^	^***^	^***^	^***^
Wounding stress x storage time			^***^	^***^	^***^	^***^	^***^	^***^
Wounding stress x water stress x storage time			^***^	^***^	NS	^***^	^***^	^***^

i *Concentrations are reported as chlorogenic acid equivalents for 3,5-diCQA, 3-hCQA, 4,5-diCQA, and IC; as gallic acid equivalents for GAD; as p-coumaric acid equivalents for p-CAD and as ferulic acid equivalents for FAD*.

ii *Compounds were quantified at 280 nm (PCA, GAD, and IC) and at 320 nm (CHA, 3,5-diCQA, 3-hCQA, 4,5-diCQA, p-CA, FA, p-CAD, and FAD)*.

iii *Data represents the mean of 3 replicates ± standard error of the mean*.

iv *Different letters in the same column indicate statistical difference by the LSD test (p < 0.05), ND, Not detected*.

v *Asterisks indicate that main effects and interactions are significantly different by analyses of variance (ANOVA), NS, non significant*;

* *p < 0.05*;

**p < 0.01;

*** *p < 0.001*.

*PCA, protocatechuic acid; GAD, gallic acid derivative; CHA, chlorogenic acid; 3,5-diCQA, 3,5-dicaffeoylquinic acid; 3-hCQA, 3-hydroxy dihydro chlorogenic acid; 4,5-diCQA, 4,5-dicaffeoylquinic acid; p-CA, p-coumaric acid; FA, ferulic acid; p-CAD, p-coumaric acid derivative; FAD, ferulic acid derivative; IC, isocoumarin. Compounds were identified and quantified by HPLC-PDA*.

Wounding stress induced the accumulation of most phenolic compounds with the exception of *p*-CA and GAD, where wounding stress provoked a complete depletion of *p*-CA and a gradual decrease in concentration of GAD. CHA and IC contents increased during storage of the wounded tissue, showing ~500 and ~1300% highest levels at 48 h of storage, respectively, as compared with wholes before storage.

Water stress exerted a different effect on the accumulation of each phenolic compound during storage of wounded carrots, inducing increments in PCA (~50%), GAD (~3%), 3,5-diCQA (~20%), 3-*h*CQA (~30%), 4,5-diCQA (~22%), *p*-CAD (~12%), FAD (~5%), and decrements in CHA (~30%), FA (~10%), and IC (~60%) at 48 h of storage as compared with wounded samples under control conditions. Likewise, while the *p*-CA was not detected in the wounded tissue, water stress induced the accumulation of this compound in shredded carrots (Table 4).

## Discussion

### Effect of water and wounding stress on the primary metabolism

The application of water stress affected the expression of genes related with the primary metabolism (DAHP synthase, EPSP synthase, and CMPD) as well as the accumulation of primary metabolites (shikimic acid and L-phe) needed as carbon source for phenolics and lignin biosynthesis (Tables 1, 2). DAHP synthase and EPSP synthase genes encode for enzymes involved on the shikimic acid pathway where chorismate, the precursor of L-phe, is produced (Bentley, 1990; Herrmann and Weaver, 1999). On the other hand, CMPD gene encode for a bi-functional enzyme that catalyzes the first committed step in L-phe biosynthesis, which includes the conversion of chorismate to prephenate and the decarboxylation and dehydration of prephenate to phenylpyruvate (Maeda and Dudareva, 2012). Although the stress induced expression of genes encoding enzymes of the shikimate pathway has been mainly studied in the context of plant-pathogen interactions (Görlach et al., 1995; Eberhard et al., 1996; Mobley et al., 1999) *in-silico* analysis and expression profiling in rice and *Arabidopsis* under drought stress suggests slight changes on the expression of EPSP synthase (Garg et al., 2014).

The accumulation of primary metabolites (shikimic acid and L-phe) was also influenced by the application of water stress (Table 2). As earlier described, shikimic acid accumulated during the first 12 h of storage in whole carrots showing a higher accumulation those samples treated with water stress (Table 2). Similar observations were reported for *Eucalyptus* (Warren et al., 2012) and maize (Sicher and Barnaby, 2012), where plants grown under water stress showed higher levels of shikimic acid. Although higher accumulation of shikimic acid was observed at 12 h of storage in water stress treated wholes, the relative expression of DAHP synthase gene, encoding the enzyme that catalyzes the first step in the shikimic acid pathway, was lower than 1.0 during the first 12 h of storage. This observation suggests that although DAHP synthase gene expression decreased as compared to time 0 h samples, the increased levels of shikimic acid observed at 12 h of storage in water stress treated wholes could be related to a direct water stress induced activation of DAHP synthase and other enzymes (3-dehydroquinate synthase and 3-dehydroquinate dehydratase) related with shikimic acid biosynthesis. Likewise, at 12 h of storage the relative expression of EPSP synthase and CMPD genes (involved on the shikimic acid conversion into downstream metabolites needed for aromatic amino acids biosynthesis) was lower in water stress treated wholes as compared to control conditions, in agreement with the higher levels of L-phe detected in the control at 12 h of storage (Table 2).

From 12 to 36 h of storage, shikimic acid content continued to be higher in water stress treated wholes, however its concentration rapidly decrease after 12 h in both samples, and at 48 h of storage no significant difference in shikimic acid content was detected between the control and water stress treated samples. Likewise, during storage of whole carrots, L-phe content was lower in wholes treated with water stress as compared to the control. The substantial shikimic acid decrements observed after 24 h of storage in wholes under water stress, and the lower L-phe levels quantified in these samples, indicates that during storage the production of metabolites needed as a defense toward water stress (i.e., lignin, Figure 2B) was intensified by such stress condition. Therefore, shikimic acid and L-phe utilization rates were higher than their biosynthesis rates in water stress treated wholes as compared to the control. This observation agrees with the relative expression detected for the three primary metabolism related genes evaluated (DAHP synthase, EPSP synthase, and CMPD), which was higher in water stress treated wholes as compared to controls at 48 h of storage, suggesting that the primary metabolism is activated by water stress as a mechanism to support the biosynthesis of secondary metabolites required to produce lignin.

Regarding the effect of wounding stress on the primary metabolism, overall, shredded carrots showed higher relative expression of DAHP synthase, EPSP synthase, and CMPD (Table 1). The wound-induced activation of DAHP synthase and chorismate mutase has been previously reported in *Brassica juncea* seedlings (Sharma et al., 1999), *Arabidopsis thaliana* (Keith et al., 1991), carrots (Jacobo-Velázquez et al., 2015), tomato and potato (Dyer et al., 1989). As earlier described, the wound-induced activation of primary metabolism related genes was enhanced when shredded carrots were treated with additional water stress. Although this is the first report in literature evaluating the combined effect of wounding and water stress on the primary metabolism of plants, a previous report in *Arabidopsis* indicates that many wound-inducible genes are also activated by dehydration (Reymond et al., 2000). Therefore, the higher relative expression of primary metabolism related genes observed in wounded carrots treated with water stress could be related to the pivotal effect that water stress exerts during the plant response to wounding (Reymond et al., 2000).

Wounded carrots showed higher levels of shikimic acid during storage as compared to wholes, showing its highest levels at 24 h of storage (Table 2). Likewise, wounding stress induced a decrement in L-phe content (Table 2). The wound-induced accumulation of shikimic acid in carrots has been previously reported (Becerra-Moreno et al., 2012). This is the first study reporting the effect of wounding on L-phe content in plants. The higher shikimic acid content obtained in wounded plants as compared to whole, match with the higher relative expression of DAHP synthase observed in shredded carrots. However, although CMPD gene was induced by wounding stress, lower L-phe levels were quantified in shredded carrots, suggesting that although L-phe biosynthesis was wound-induced its utilization rate for stress metabolites biosynthesis was higher in wounded carrots, avoiding its accumulation. These observations match with the increases in total phenolics and lignin content detected during storage of wounded carrots (Figure 2).

Regarding the combined effect of wounding and water stress on the accumulation of primary metabolites, although the expression of genes related with their biosynthesis was enhanced by the application of water stress in shreds, wounded tissue treated with water stress showed lower levels of shikimic acid and no significant difference in L-phe content was observed between both samples during storage (Table 2). These results imply that although the production rate of these metabolites were enhanced by the application of water stress in the wounded tissue, their conversion rate into water stress related metabolites such as lignin was also increased (Figure 2B), and thus no accumulation was observed. To the best of our knowledge this is the first report in literature evaluating the combined effect of wounding and water stress on the accumulation of L-phe and shikimic acids in plants.

There is limited scientific information on the molecular mechanisms regulating the wound and water stress induced activation of the primary metabolism in plants. However, it is well known that wounding and water stresses induce ethylene (Lurie et al., 1986; Burdon et al., 1994; Kubo et al., 2000) and reactive oxygen species (ROS) production (Kranner, 2002; Kranner et al., 2002) which are stress-signaling molecules that activate the primary and secondary metabolism of plants (Orozco-Cárdenas et al., 2001; Song et al., 2006; Jacobo-Velázquez et al., 2011, 2015; Jacobo-Velázquez and Cisneros-Zevallos, 2012). Likewise, the dramatic decrease of L-phe content observed in wounded carrots could also be acting in combination with ethylene and ROS as a signal to active genes related with its biosynthesis (O'Donnell et al., 1996; Orozco-Cárdenas et al., 2001; Watanabe et al., 2001).

### Effect of water and wounding stress on the secondary metabolism

The application of water stress affected the expression of genes related with the biosynthesis of hydroxycinnamic acids and lignin (Table 3), as well as the accumulation of these metabolites (Figure 2, Table 4). As observed for genes involved in the primary metabolism (Table 1), water stress up regulated the expression of secondary metabolism related genes. Carbon flux through the phenylpropanoid pathway in plants requires the sequential action of PAL, C4H, and 4CL (Kumar and Ellis, 2003). In conjunction with PAL and 4CL, C4H directs carbon flux to an array of important phenolic compounds in plants including lignin, suberin, flavonoids, and numerous other phenylpropanoids. Likewise, CCoAOMT, CCR, and CAD are involved on the biosynthesis of monolignols, which are precursors of lignin (Ye et al., 1994; Boerjan et al., 2003). The water stress induced activation of genes related with the biosynthesis of phenolic compounds and lignin has been previously reported in plant tissues such as rice (Yang et al., 2006), maize (Fan et al., 2006), and watermelon (Yoshimura et al., 2008). Although wholes treated with water stress showed higher relative expression of genes related with the biosynthesis of phenolic compounds and lignin, herein accumulation of lignin was only detected, whereas phenolic compounds decreased in water stress treated wholes (Figure 2B). Since phenolic compounds are used as precursors for lignin biosynthesis, it is likely that phenolic biosynthesis is lower than lignification process in wholes treated with water stress, hampering phenolics accumulation (Figure 2A). The results obtained herein show that lignin is synthesized as a defense mechanism to prevent water loss in plants under water stress, as has been previously demonstrated in plant tissues such as maize (Fan et al., 2006), white clover (Lee et al., 2007), and rapeseed (Lee et al., 2014).

The main individual phenolic compounds identified in carrots (Table 4) were similar to those previously reported (Surjadinata, 2006; Heredia and Cisneros-Zevallos, 2009a,b; Jacobo-Velázquez et al., 2011; Becerra-Moreno et al., 2012; Surjadinata and Cisneros-Zevallos, 2012). Interestingly, this is the first study reporting the tentative presence of 3-*h*CQA in plants, however, its identity should be confirmed by additional methods of structure elucidation such as nuclear magnetic resonance (Mateil et al., 2012). CHA content in wholes treated with water stress dramatically decreased during storage (Table 4), showing higher decreases wholes treated with water stress. CHA is one of the main precursors of lignin (Rittinger et al., 1987). Therefore, this result implies that CHA conversion into lignin was increased when whole carrots were stored under water stress conditions. Likewise, the lower levels of FAD detected in water stress treated samples could be also related to the same phenomenon, since ferulic acid is also used as a precursor of lignin biosynthesis (Boerjan et al., 2003).

In relation to the effect of wounding stress on the secondary metabolism of carrots, shredded carrots showed higher relative expression of genes related with the phenylpropanoid metabolism as compared to wholes (Table 3). The wound-induced activation of PAL, C4H, 4CL, CCoAOMT, CAD, and CCR has been previously reported in plants such as poplar (Chen et al., 2000), lettuce (Campos et al., 2004), *Arabidopsis* (Lee et al., 1995; Soltani et al., 2006), and carrots (Jacobo-Velázquez et al., 2015). The increased PAL gene expression observed in wounded carrots resulted on the accumulation of phenolic compounds during storage (Figure 2A). Likewise, the accumulation of lignin was observed in wounded carrots, matching with a higher relative expression of CCoAOMT, CAD, and CCR in the wounded tissue as compared to wholes (Figure 2B).

Wounding stress induced the accumulation of CHA and its derivatives 3,5-diCQA and 4,5-diCQA. The wound-induced accumulation of CHA was hampered when water stress was applied on the wounded tissue (Table 4). This may be attributed to the higher lignification process observed in water stress treated shreds (Figure 2B). Likewise, the accumulation of CHA derivatives was enhanced when water stress was applied on the wounded tissue, suggesting that the lower CHA levels observed in water stress treated samples could also be attributed, at least in some degree, to its conversion into 3,5-diCQA, 4,5-diCQA, and of 3-*h*CQA. A similar behavior was observed for IC content in carrots treated and non-treated with water stress (Table 4), which levels were wound-induced but water stress treated shreds showed a lower accumulation. The lower IC levels detected in water stress treated shreds could be related with lower carbon availability for its biosynthesis, since in water stress treated samples the carbon flux was directed into the production of lignin instead of other phenylpropanoids derivatives.

Regarding the combined effect of water and wounding stress on the expression of genes related with the primary metabolism and the biosynthesis of phenolics and lignin, the application of water stress produced a synergistic effect on their wound-induced activation. This synergistic effect is evident even as early as 12 h after the application of water stress conditions. As observed in Figure 1 and Table 3, wholes and shreds controls under similar moisture content (84.70 and 85.86%) showed PAL relative expression values of 3.58 and 253.77, respectively, activation that is attributed to the sole effect of wounding. However, when waters stress was applied to shreds, moisture content decreased to 81.23% and PAL relative expression increased to 3662.88, demonstrating the synergistic effect when considering that wholes treated with water stress (moisture content = 82.57%) did not show significant difference in the relative expression of PAL as compared to wholes control. Similar synergistic response was observed for all genes evaluated (Tables 1, 3). To our knowledge this is the first report on synergistic effects of water and wounding stress response in plants.

Interestingly, despite the higher relative expression of PAL detected on water stress treated shreds, total phenolic content reached lower levels as compared to wounded controls. This may be attributed to the higher lignification process inferred by the gene expression levels (CCoAOMT, CCR, and CAD, Table 3) and the lignin accumulation (Figure 2B) in water stress treated samples. As earlier described, it has been suggested that water stress exerts a pivotal effect on the wound response in plants (Reymond et al., 2000). Therefore, if wounded carrots are subjected to additional water stress it would be expected to intensify the wound-response in plants. However, in the case of wounded carrots treated with water stress, lignification processes was favored rather than phenolics biosynthesis and thus lower phenolic compounds and higher lignin content was quantified as compared to wounded carrots stored under control conditions.

### Combination of water and wounding stress as a tool to increase high value metabolites in carrot

**Figure 3:**
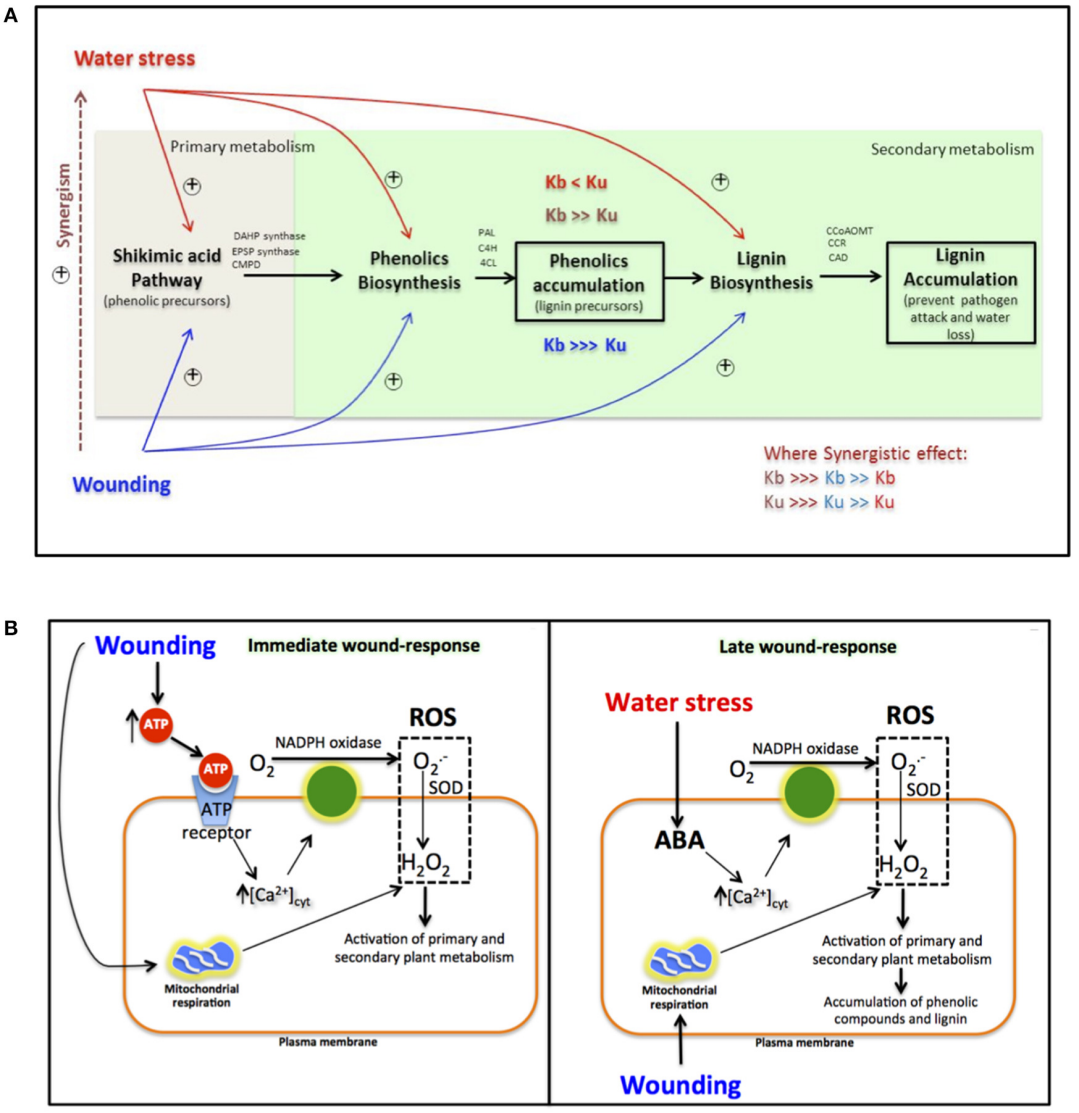
**Effect of water and wounding stress on the gene modulation of primary and secondary metabolic pathways associated with the biosynthesis of phenolic compounds in carrots (A)**. Phenolic biosynthesis rate (Kb) and phenolic utilization rate (Ku), associated to lignin biosynthesis for the prevention of pathogen attack and water loss, define the accumulation or decrease of phenolic compounds. Activation of metabolic pathways by water stress and wounding stress, are indicated by red and blue, respectively, while synergistic effects are indicated by brown color. Hypothetical model integrating wounding and water stress signaling pathways in carrots **(B)**. The integration of both signaling pathways (wounding and water stress) at the late wound-response, could result on an exacerbated ROS production, which could be responsible of the synergistic effect observed **(A)** on the wound-response of carrots treated with additional water stress. ROS, Reactive oxygen species; SOD, superoxide oxidase; ABA, abscisic acid.

The results obtained herein represent an important contribution to the understanding of the direct effect that water stress exerts on the activation of primary and secondary metabolic pathways related with the biosynthesis of phenolic compounds, when applied alone or in combination with wounding stress (Figure 3A). When carrots are subjected to water stress, the primary (shikimic acid pathway) and secondary (phenylpropanoid pathway) metabolism of plants is activated. However, although the synthesis of primary metabolites and phenolics is induced by water stress, their conversion rate into lignin is higher and thus slight accumulation of L-phe and decreases in total phenolics are observed when samples are treated with water stress alone. Wounded plants are naturally under water stress, which is a pivotal mechanism of the wound-response. However, wounding stress induces a much higher activation of these metabolic pathways compared with water stress alone. The accumulation of phenolic compounds in plants is a result of their biosynthesis rate (Kb) and utilization rate (Ku) for lignin biosynthesis (Reyes et al., 2007). If Kb > Ku, accumulation of phenolic compounds will be observed. In the case of plants treated with wounding stress, where water stress occurs naturally, Kb is higher than Ku, and thus accumulation of phenolics are observed. When water stress is applied in wounded plants both Kb and Ku are increased due to a synergistic effect. However, under such conditions Ku increases in a higher degree favoring the formation of lignin while decreasing the wound-induced accumulation of phenolics (Figure 3A).

Further experiments should be focused on understanding the physiological and molecular mechanisms involved on the synergistic effect that water stress exerts on the wound response in carrots. Based on previous work from our group and other reports, a hypothetical model showing the integration of wounding and water stress signaling pathways is proposed (Figure 3B). ROS production induced by wounding and water stress signaling pathways could be the answer to the synergistic effect that water loss exerts on the wound-response in carrots (Figure 3B). At the immediate response of wounding cytosolic ATP liberated from injured cells, initiates ROS production (Song et al., 2006). This ROS production is catalyzed by NADPH oxidase, which activation is mediated by an increase of cytosolic Ca^2+^ concentration after ATP binding to its receptor. Superoxide radical (O${}_{2}^{.-}$) produced by NADPH oxidase, is then transformed into H_2_O_2_ by superoxide dismutase (SOD). Simultaneously, wounding produces an immediate increase in mitochondrial respiration, which represents an additional source of ROS (Jacobo-Velázquez et al., 2011). Previous reports suggest that this immediate burst of ROS after wounding is responsible of the activation of primary and secondary metabolic pathways involved on phenolics biosynthesis (Razem and Bernards, 2003; Jacobo-Velázquez et al., 2015). As a late response to wounding (Figure 3B), plants start to experience water stress, and produce abscisic acid (ABA) (Lulai and Suttle, 2009). ABA also triggers NADPH oxidase ROS production by increasing cytosolic Ca^2+^ concentration (Cho et al., 2009). Therefore, ABA produced, as a response to water stress in wounded plants in addition to mitochondrial respiration could be responsible, at least partially, of the second burst of ROS previously reported for wounded plants (Razem and Bernards, 2003; Jacobo-Velázquez et al., 2011). This second burst of ROS has been associated with triggering the production of suberin and lignin during the wound-healing process (Razem and Bernards, 2003; Lee et al., 2013). Therefore, the integration of both signaling pathways (wounding and water stress) at the late wound-response, could result on an exacerbated ROS production, which could be responsible of the synergistic effect observed on the wound-response of carrots treated with additional water stress.

It is important to point out that the response to the abiotic stresses evaluated herein may be different depending on the plant organ and if the stress is applied during pre- or post-harvest, and thus further experiments should be focused on validating the hypothetical model shown on Figure 3B. Likewise, in the present experiment the control samples for wounding (with no additional water stress applied) were stored under ~35% of relative humidity, and thus were under mild water stress conditions. Therefore, future studies evaluating the physiological response of plants to wounding stress applied alone or in combination with waters stress should consider storing the wounded tissue under ~95–100% of relative humidity in order to minimize the natural water stress conditions occurring in wounded tissue.

## Conclusions

In the present study, it was demonstrated that water stress is one of the pivotal mechanism of the wound-response in carrot. Water stress activated the primary and secondary metabolism of carrots, favoring the lignification process. Furthermore, wounding stress induced higher activation of the primary and secondary metabolism of carrots as compared to water stress alone, leading to higher accumulation of shikimic acid, phenolic compounds, and lignin. Moreover, the wound-induced activation of primary and secondary pathways related with the biosynthesis of phenolic compounds, was synergistically enhanced when the wounded tissue was treated with additional water stress, however since the lignification process was increased, lower accumulation of phenolic compounds was detected. Results allowed the elucidation of strategies to induce the accumulation of specific primary or secondary metabolites when plants are treated with water stress alone or when additional water stress is applied on wounded tissue. If the accumulation of a specific primary or secondary metabolite were desirable, it would be recommended to apply both stresses to accelerate their biosynthesis. However, strategies such as the use of enzymatic inhibitors to block the carbon flux and enhance the accumulation of specific compounds should be designed. For instance, if the accumulation of shikimic acid is desirable, it would be suggested to apply glyphosate (inhibitor of EPSP synthase) to wounded carrots stored under additional water stress, as previously reported by Becerra-Moreno et al. (2012). Likewise, it is likely that the application of PAL inhibitors (i.e., 2-aminoindan-2-phosphonic acid, α-aminooxyacetic acid, and α-aminooxi-β-phenylpropionic acid) and lignin biosynthesis inhibitors (i. e., N-(O-hydroxyphenul)-and N-(O-aminophenyl)sulfinamoyltertiobutyl acetate) in plants treated with wounding and water stress would lead on the accumulation of high levels of L-phe and phenolic compounds, respectively.

## Author contributions

AB, and DJ designed experiments. AB, MR, and VN, carried out experiments. AB, MR, and VN, processed data. AB, JB, LC, and DJ, analyzed data and wrote the main text of the manuscript. All authors read and approved the final manuscript.

### Conflict of interest statement

The authors declare that the research was conducted in the absence of any commercial or financial relationships that could be construed as a potential conflict of interest.
