# Supplementary material for: Combined effect of water loss and wounding stress on gene activation of metabolic pathways associated with phenolic biosynthesis in carrot
Source: Front Plant Sci. 2015 Oct 15;6:837. doi: 10.3389/fpls.2015.00837 (PMC4606068; doi:10.3389/fpls.2015.00837)
Supplement: Supplementary file 1 [file Table1.PDF]

Table S1. Sequences of primers used for real-time quantitative reverse transcription PCR (qRT-PCR) analyses.

**Putative gene identification (Accession number)**	**Sequence for forward (F) and reverse (R) primers (5'→3')**
Primary metabolism genes	
Shikimate pathway genes	
3-deoxy-D-arabino-heptulosonate (KT280466)	F: GATGCTATCAGGGCTGAAGTCAA<br>R: CCTAGATCATCAAAAGTCACGGTTC
5-enolpyruvylshikimate 3-phosphate synthase (KT280467)	F: TAGGAGCAACAGTTGAAGAGGGT<br>R: ATCTGAACAGGCAGCAAGAGAGA
Aromatic amino acid biosynthesis genes	
Chorismate mutase-prephenate dehydratase (KT280468)	F: CGAGCACACGTTGACGAAGCTA<br>R: AAATTCCGCAGCTCCAGCAGT
Secondary metabolism genes	
Hydroxycinnamic acids biosynthesis genes	
Phenylalanine ammonia-lyase (BAA23367)	F: AGGGCAAACCCGAATTTACTGAC<br>R: CATAAGAGCTTCCATCCAAGATG
Trans-cinnamate 4-monooxygenase (AIT52342)	F: CCTGGAATCATCCTTGCATTGC<br>R: TGTCCTCCTTTCTCTGCTGTGT
4-coumarate-coa ligase (AIT52344)	F: GGGACTACTGGGACTTCCAAAG<br>R: AGCCAAATAAACCATCTCCCAGC
Lignin biosynthesis genes	
Caffeoyl-CoA 3-O-methyltransferase (KT280469)	F: TATTCTCTCCTTGCCACTGCTCT<br>R: CCCTCTCTGAAGTCAATTTTGTG
Cinnamoyl-CoA reductase (KT280470)	F: AATCCCTCAGCAGCAGGAAGATA<br>R: TTGGTAGGCACGGGGTATTCAG
Cinnamyl alcohol dehydrogenase (KT280471)	F: GGAGGCAATGGATCATCTTGGT<br>R: GAGAGGTAAGGTTCGAGTGGGT
