# Supplementary material for: Combined effect of water loss and wounding stress on gene activation of metabolic pathways associated with phenolic biosynthesis in carrot
Source: Front Plant Sci. 2015 Oct 15;6:837. doi: 10.3389/fpls.2015.00837 (PMC4606068; doi:10.3389/fpls.2015.00837)
Supplement: Supplementary file 2 [file Table2.PDF]

Table S2. Effects of wounding and water stress on total phenolic and total lignin content in carrots.

.

Sample	Storage time	Storage conditions	Total phenolics (mg/kg DW) [i]	Total lignin (mg/kg DW) [i]
	0 h	Control	2103.64 ± 132.19 g	31748.35 ± 744.50 l
Wholes	12 h	Control	1367.32 ± 115.06 h, i	38780.74 ± 1266.44 k
		Water stress	1325.08 ± 126.57 h, i	65376.02 ± 1285.33 h
	24 h	Control	1226.77 ± 118.42 h, i	32405.80 ± 739.19 l
		Water stress	946.03 ±114.04 i	52370.67 ± 1330.84 j
	36 h	Control	1545.80 ± 112.86 h	32301.82 ± 635.10 l
		Water stress	1128.25 ± 103.84 h, i	52942.87 ± 1617.99 j
	48 h	Control	999.18 ± 119.06 i	28249.06 ± 852.91 m
		Water stress	1136.37 ± 105.82 h, i	59750.80 ± 2384.53 i
Shreds	12 h	Control	3068.71 ± 149.99 f	72506.63 ± 1426.94 f, g
		Water stress	3047.44 ± 141.71 f	71254.67 ± 1372.35 g
	24 h	Control	5872.54 ± 268.29 c	73728.61 ± 1924.53 f
		Water stress	4419.60 ± 240.32 e	88574.51 ± 2444.25 d
	36 h	Control	6949.66 ± 408.17 b	85431.63 ± 2798.73 e
		Water stress	5175.82 ± 248.62 d	102441.19 ± 2755.48 b
	48 h	Control	9690.39 ± 332.61 a	94264.36 ± 3298.03 c
		Water stress	6676.16 ± 180.23 b	115700.31 ± 3551.48 a
**Significance** [ii]				
Wounding stress			***	***
Water stress			***	***
Storage time			***	***
Wounding stress x water stress			*	***
Water stress x storage time			***	***
Wounding stress x storage time			**	***
Wounding stress x water stress x storage time			**	***

(i) Values represent the mean of 3 replications ± standard error of the mean. Different letters in the same column indicate statistical difference by the LSD test ($p < 0.05$). (ii) Asterisks indicate that main effects and interactions are significantly different by ANOVA. NS – non significant, $^{*}p$<0.05, $^{**}p$<0.01, $^{***}p$<0.001.
