# Supplementary figures and images for: Combined effect of water loss and wounding stress on gene activation of metabolic pathways associated with phenolic biosynthesis in carrot
Source: Front Plant Sci. 2015 Oct 15;6:837. doi: 10.3389/fpls.2015.00837 (PMC4606068; doi:10.3389/fpls.2015.00837)

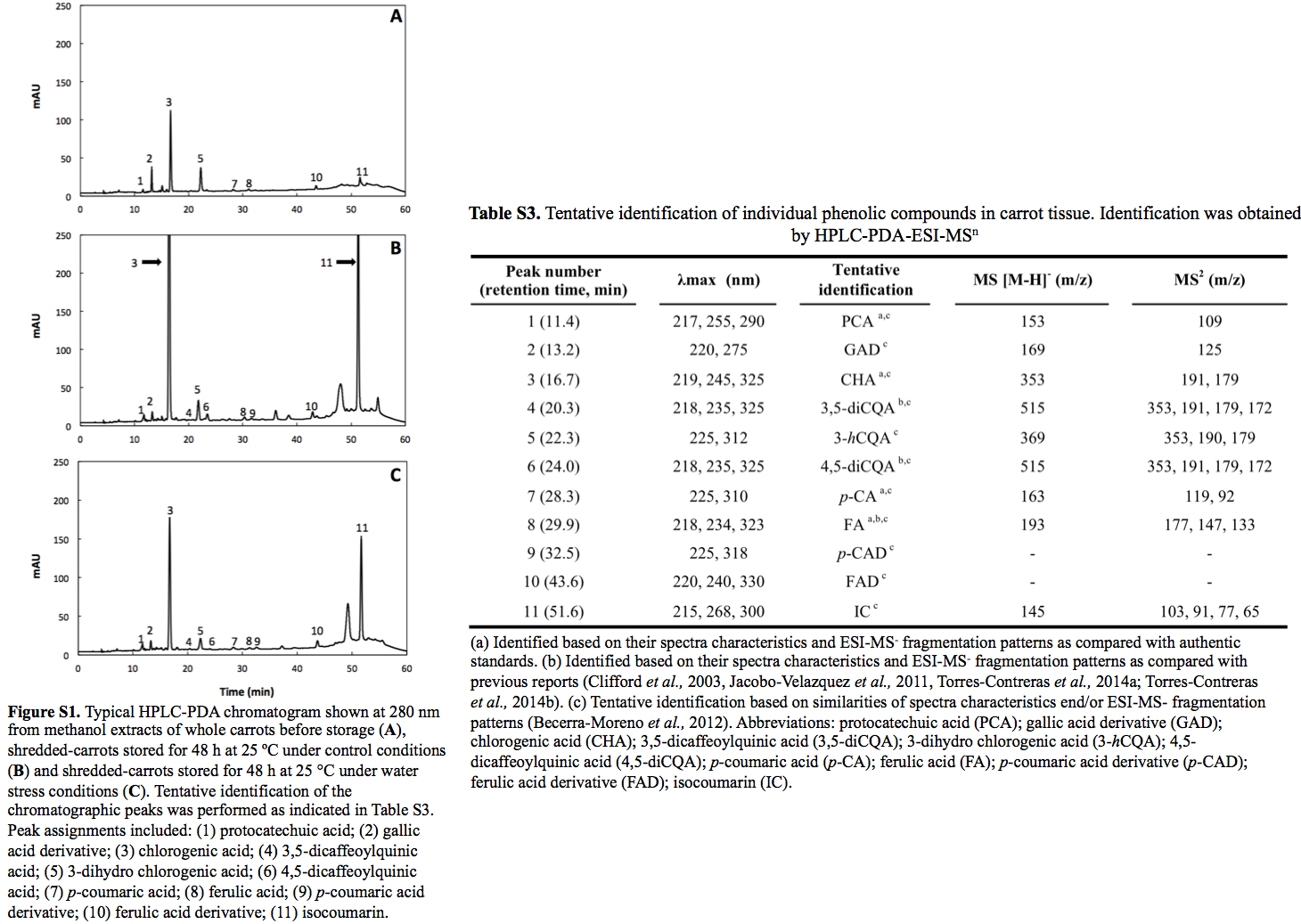

Supplement: Supplementary file 3 [file Image1.TIFF]
